# The Applicable Dental Age Estimation Methods for Children and Adolescents in Indonesia

**DOI:** 10.1155/2022/6761476

**Published:** 2022-02-15

**Authors:** Arofi Kurniawan, An'nisaa Chusida, Nur Atika, Tito Krisna Gianosa, Mohammad Denis Solikhin, Mieke Sylvia Margaretha, Haryono Utomo, Maria Istiqomah Marini, Beta Novia Rizky, Beshlina Fitri Widayanti Roosyanto Prakoeswa, Aspalilah Alias, Anand Marya

**Affiliations:** ^1^Department of Forensic Odontology, Faculty of Dental Medicine Universitas Airlangga, Surabaya, Indonesia; ^2^Department of Basic Sciences and Oral Biology, Faculty of Dentistry, Universiti Sains Islam, Nilai, Malaysia; ^3^Department of Orthodontics, Faculty of Dentistry, University of Puthisastra, Phnom Penh, Cambodia; ^4^Center for Transdisciplinary Research, Saveetha Dental College, Saveetha Institute of Medical and Technical Science, Saveetha University, Chennai, India

## Abstract

Indonesia is an archipelagic country bordered by tectonically active zones with intense seismicity and volcanism. This condition is often associated with a high-risk situation of disasters in Indonesia. Forensic identification is a necessary procedure to reveal an individual's identity. An identity, including sex and age, is needed to build a conclusion of human identification. Dental age estimation is a subfield of forensic odontology which focuses on establishing an individual's age. Tooth development, biochemical, and postformation changes are the parameters for estimating dental age. This review discusses the applicable dental age estimation method for children and adolescents in Indonesia. Several articles that have previously studied dental age estimation in Indonesia were reviewed for this manuscript. On reviewing these articles, it was found that the Demirjian method, the Willems method, and the Al Qahtani method are useful in this population with higher accuracy than other methods.

## 1. Introduction

Indonesia is an archipelagic country located in the *ring of fire* area. It is bordered by tectonically active zones characterized by intense seismicity and volcanism. This geographic condition is often associated with the high risk of disaster in Indonesia. Many natural disasters such as earthquakes, volcanic eruptions, tsunamis, and liquefaction have happened in Indonesia. Besides, terrorism, airplane accident, traffic accident, and criminal cases often result in many victims. Based on the national disaster management board (BNPB Indonesia), 4.650 disasters happened during the 2020 period [1].

Identification of the victim in a disaster situation is challenging for the DVI team. Many identification methods can be applied to establish human identities, such as visual identification, DNA analysis, fingerprint analysis, and dental findings. The basic principle of identification is the comparison between the antemortem data and postmortem data [2]. DNA analysis provides high accuracy for identification; however, it needs high costs and is time-consuming. Postmortem fingerprints will be matched with fingerprint databases. Postmortem dental findings can be compared with the antemortem dental records from the dentist [3].

Teeth are the most durable part of the human body that can survive in extreme conditions. Much important information, including sex, age, ethnicity, and social status, can be obtained from human teeth. Age is an essential aspect of human life. The biological age can be estimated using several parameters in forensic sciences, such as bone and teeth. Tooth development and eruption, postformation changes, and the third molar are the parameters for estimating the dental age. Various dental age estimation methods have been developed in many studies. Each method has different accuracies and is limited to a specific population [4, 5]. The present study discussed the applicable dental age estimation method for children and adolescents in Indonesia.

## 2. Tooth Growth and Development

The structure of the growth and development of teeth occurs under strict genetic control to determine the different tooth morphology in each human. Tooth development is regulated through the stimulating interactions of epithelial cells and mesenchyme. This interaction is for a single mechanism in the process of organogenesis. Epithelial-mesenchymal interactions control cell numbers by stimulating mitosis, preventing apoptosis near the surface of the tissue, affecting cell shape, cell differentiation, and stimulating the release of several molecules. The growth and development of teeth go through a complex process such as starting from the initiation process. This process shows the development of the jaw, where the teeth will grow and develop, and is associated with the morphogenesis pattern. The thickening of the oral epithelium develops into a cap-like structure covering the dental papilla's mesenchymal cells, which then form the odontoblast and dental pulp. Then, the formation of the crown morphology occurs during the bell stage; as the cusp develops, the mesenchymal cells cooperate with the epithelium during the early stages of tooth formation. In addition, the enamel knot acts as the center for marking and regulating the shape of the tooth bulge. There is also growth in the speed of the cell death to show the differences in the shape and size of the tooth cusp.

The expression of the BMP family also plays a role in tooth development and is present in differentiated cells. Mesenchyme cells work together with the epithelium during the early stages of tooth formation. In addition, the enamel knot acts as the center for marking and regulating the shape of the tooth bulge. There is also the growth in the speed of the cell death to show the differences in the shape and size of the tooth cusp. The expression of the BMPs family also plays a role in tooth development and is present in differentiated cells [6–8].

The process of growth and development of teeth is very complex, and they contain detailed information about individuals because their characteristics are different for each individual. In addition to the characteristics possessed by teeth, teeth also have a functional role such as in the masticatory system, as an indication of other or systemic diseases, and they have a major role in determining the estimated age of a person [9].

### 2.1. Dental Age Estimation

Dental maturity has an essential role in children's and adolescents' age estimations [10]. The number and eruption sequence of the teeth can determine the age of an individual [11]. Teeth are the hardest body structure, resistant to external influences, and undergo the most minor biological changes. In addition, teeth can also provide information about an individual's identity because of their unique characteristics. This is because teeth have a stage of tooth growth and development as an indicator of age estimation, controlled more by genetic factors. Therefore, dental age shows minor variation compared to bones and other body parts [9, 12]. The accuracy of the results obtained from the age estimation depends on the method used. This consists of clinical, radiographic, histological, and biochemical methods [13]. The radiographic method is the most widely used because of its applicability in living and dead individuals and specific judicial setups that prohibit tissue collection from human remains because it uses a dental radiograph, hence eliminating the requirement to extract the teeth [14].

Age estimation plays an essential role in the identification process of victims of natural disasters and airplane accidents. Because not a few of these cases have claimed victims whose conditions have been damaged and who have lost their identities. Age can be estimated because of increasing age and the subsequent development of body structures which in turn can be related to an individual's age [15, 16].

The choice of the age estimation method to be used must consider several factors. This is so that the method to be used is under the circumstances of each case. Living individuals can use any method, but according to their age, in contrast to dead individuals, they must go through the teeth using all examination methods, namely, clinical, radiographic, histological, and biochemical. This is because the dead individuals' teeth can be extracted. In addition, the age category of an individual must also be considered in choosing an age estimation method; the age categories are as follows:

1. Only radiographs can be used at prenatal age because at this age, we can only see and assess the stage of formation and development of unerupted primary teeth. Methods used include the Schour and Masseler Atlas method, the Al Qahtani Atlas method, the Blenkin–Taylor Atlas method, Gustafson's method, and the Koch diagram.
2. Children and adolescents at this age can use radiographic examination methods and clinical examination by counting the number of teeth that have erupted, for example, at the age of 6 months to 2.5 years. The radiographic examination can also be performed at the age of 6 months to 16 years, with the Demirjian scoring method and the open apical method by Cameriere at the age of 3 to 16 years. Furthermore, at the age of 17 to 23 years, third molar development methods such as the Harris and Norje methods can be used.
3. Adult age, the adult age category (21 years and over), can be examined for changes in tooth structure and the development of third molar teeth. The appropriate method of estimating an individual's age is histological examination by looking at changes in tooth structure as described by Gustafson and Johanson [17].

### 2.2. Demirjian Method

In 1973, Demirjian introduced a method to estimate chronological age based on tooth development. This method makes use of the seven left permanent mandibular teeth except for the third molar. The Demirjian method describes 8 stages of tooth calcification ranging from stage A to stage H, and stage 0 means that there is no tooth calcification seen in the panoramic photo. This method assessed the central incisors, lateral incisors, canines, first premolars, second premolars, first molars, and second molars. Not only that, the scoring system for this method is also differentiated between boys and girls [18, 19].

Determining the chronological age, especially the age of children, has a high level of difficulty when viewed from the point of view of tooth eruption. Therefore, the best way is to assess the age of the teeth through the stages of tooth development. Like the Demirjian method, this is one of the methods used to measure the maturation stage of teeth from 3 to 17 years of age. This method is shown to have high accuracy and highly correlates with chronological age. But this method has deficiencies in determining the exact age due to variations in each individual. This is due to various factors such as the method used, the accuracy of the observer, the number of samples, and the distribution [20]. According to Puranik and Uma (2015), the Demirjian method is considered a simple and reliable method as it is a panoramic radiograph-based method with good reproducibility and reliable standardization [21].

In the study by Yunus and Wardhani in 2016, 30 panoramic radiograph samples of Indonesian children between 4–9 years old were calculated using the Demirjian method. The result showed that the Demirjian method is less accurate because there is a significant overestimation of dental age compared with the chronological age [22].

### 2.3. Willems Method

Willems's method is a modified Demirjian method. Willems et al. modified the Demirjian method after knowing that this method overestimated age in the Belgian Caucasian population. This adapted method later became known as the Willems method [23, 24]. According to Willems, this method can result in more accurate estimation results than the Demirjian method in the child population [25]. This method assesses the degree of development of each of the 7 teeth on the left side of the mandibular except for the third molar. Each of the teeth has an individual score assigned based on the Demirjian calcification stage. The scores of all the individual teeth are added up and converted to the chronological age based on sex [26].

### 2.4. Al Qahtani Method

The Al Qahtani method is the newest method as a complement to the existing methods. This method has essential evidence, high accuracy, sensitivity, and is easy to estimate age. The Al Qahtani method requires radiographic images to assist in examining age stimulation. The radiographic image used in this method is a panoramic radiograph because it has a broad picture covering all the teeth in the maxilla and mandible [27, 28].

In the research by Rusydiana et al. in 2016, a total of 94 samples were calculated using the Al Qahtani method. The results showed that 66 (70.21%) of the samples using the method were able to get a good result of estimating the patient's chronological age, while the rest of 28 (29.79%) of the samples' estimation results were significantly different from the patient's chronological age [29].

### 2.5. TCI-Benindra Method

The TCI-Benindra method is a known method carried out for identifying age estimates through radiography, the tooth coronal index method (TCI). This method is based on the relationship between chronological age and the size of the dental pulp chamber. The size of the dental pulp chamber shows a significant correlation with the chronological age of individuals14, 16. This method uses a panoramic radiograph view of the premolars and mandibular molars (except third molars) because the panoramic radiograph shows a picture of the pulp chamber of the mandibular teeth, which is clearer than the maxillary teeth, and only pulp space in the posterior teeth, which can usually be completely clear [30].

In the study by Yulianti et al. in 2017, a total of 70 periapical radiograph samples of the Banjarese population were calculated using the TCI-Benindra method. The result showed that the value of the tooth coronal index would decrease even more when the person is getting older. This occurs due to changes in the pulp chamber caused by the formation of a layer of secondary dentin [31].

### 2.6. Kvaal Method

The Kvaal method is based on the relationship between age and tooth to pulp ratio to determine an individual's chronological age [32, 33]. In the examination using a regression formula, namely, age = 133-(313.8 × M)-(65 × [WL]). The measured dental pulp ratio will be substituted into the existing formula to determine whether or not there is a significant difference with respect to the patient's chronological age. This method uses a dental pulp ratio because secondary dentine is deposited along the pulp chamber wall, which causes a reduction in the size of the pulp chamber with age. The examination using the Kvaal method is also assisted by using radiographic images. The reason is that it is more noninvasive and saves time. However, the Kvaal method has a drawback: its incorrect accuracy in examining vulnerable patients aged 19 to 20 years. [34].

In the study by Farahyati et al. in 2018, 34 panoramic radiographs and 25 intraoral periapical, panoramic, and lateral cephalometric radiograph samples in the age group of 16–21 years old were calculated using the TCI-Benindra, Kvaal, and Schour and Massler Atlas methods. The result showed no significant difference between the TCI-Benindra method in the panoramic and periapical radiographs. There is a significant difference between TCI-Benindra on the periapical radiograph and the Kvaal method on the panoramic radiograph and a significant difference between TCI-Benindra on the panoramic radiograph and the Schour and Massler method on the lateral cephalometric radiograph [35].

### 2.7. Nolla Method

Nolla's method assesses the chronological age by evaluating the calcification of the permanent dentition. The calcification is divided into 10 stages, from the tooth seed's formation until the apical foramen closure. The formation of the crypt to the closure of the root apex of the teeth, which can be seen on the radiographic image implies level 1 and the root apex closure stage is said to be level 10 [36, 37]. Panoramic photos assist this method, and the part of the teeth to be observed is the 3rd molar, the permanent maxillary tooth, and the lower jaw, which will be stage-matched and scored. Each root score is totaled and converted using the Nolla method conversion tables [38].

In the study by Marinda et al. in 2019, a total sample of 30 panoramic X-rays with susceptible ages of 10 to 11 years was calculated with the Nolla method. The results obtained from this study showed significant differences between chronological age and the estimated outcome of age using the Nolla method. This is due to various factors, including the quality of the reference material, the reliability of the measurement method, and the biological variability in tooth development, which can cause observer differences [39].

### 2.8. Schour and Massler Method

Schour and Massler studied the development of the primary and permanent teeth, describing 21 chronological stages from 4 months to 21 years of age and publishing them in the form of numerical developmental diagrams. The American Dental Association (ADA) has periodically reviewed these charts and published them in 1982, making it possible to compare the degree of calcification on radiographs with the standard Schour–Massler had established [40].

## 3. Discussion

Many methods of age estimation can be used, for which various factors must be considered. This is because the accuracy and the results depend on the most appropriate method in the circumstances of each case and condition. These factors include individual status (alive or dead), individual age category, number of individuals whose age has to be identified, types of single or mass cases, availability of teeth and support networks, location of cases, availability of age estimation tools and tools, as well as culture and religion, adopted by the individual to be identified [17].

The age estimation method is based on age categories, where the Demirjian estimation method can be applied to the ages of children and adolescents. This is evidenced in the study by Sasmita et al. in 2020, which researched the age of 3 to 17 years using this Demirjian method. The results show that this method has high accuracy and is highly correlated with chronological age. Even so, this method still has a drawback; that is, it cannot determine the exact age due to variations in each individual.

The Willems method is a modified result of the Demirjian method, where the examination stage is the same as the Demirjian method, namely, using a calcification that has its score [20]. Apart from the Demirjian and Willems methods, the Al Qahtani method also has high accuracy and is the most modern method. This was proven by Rusydiana et al. [29], who examined 94 samples. Of the 94 samples tested, 66 of them got a similar result to the patient's age. This means that there are 70.21% of samples according to the patient's age. However, the Al Qahtani method also has drawbacks because it can only be examined on panoramic radiographs and other drawbacks. This method cannot be used at prenatal age, newborns, and infants.

In addition, other age estimation methods have been used in Indonesia, such as the Kvaal method and the Nolla method. However, both methods have reported less accurate results in several age categories. Like the Kvaal method, which turned out to be inaccurate accuracy on examinations with susceptible ages of 19 to 20 years, and in addition to the Nolla method, the results obtained from the study demonstrated a significant difference between the chronological age and the results obtained by age estimation. This is because it is caused by various factors such as the duality of the reference material, the reliability of the measurement method, and the biological variability in tooth development which can cause observer differences [39].

## 4. Conclusion

Many age estimation methods are applicable in Indonesia. The findings of this review indicate that the dental age estimation methods proposed by Demirjian, Willems, and Al Qahtani are the most appropriate methods for dental age estimation of children and adolescents in Indonesia. These methods show high accuracy with minimum errors and are easily applied in daily forensic work [21].

## Data Availability

All types of data, which were used to support this review are included within the article.
